# Brain tissue oxygen monitoring in traumatic brain injury: part I—To what extent does PbtO_2_ reflect global cerebral physiology?

**DOI:** 10.1186/s13054-023-04627-y

**Published:** 2023-08-31

**Authors:** Teodor Svedung Wettervik, Erta Beqiri, Stefan Yu Bögli, Michal Placek, Mathew R. Guilfoyle, Adel Helmy, Andrea Lavinio, Ronan O’Leary, Peter J. Hutchinson, Peter Smielewski

**Affiliations:** 1 Section of Neurosurgery, Department of Medical Sciences, Uppsala University, 751 85 Uppsala, Sweden; 2 Brain Physics Laboratory, Division of Neurosurgery, Department of Clinical Neurosciences, University of Cambridge, Cambridge, UK; 3 Division of Neurosurgery, Department of Clinical Neurosciences, Addenbrooke’s Hospital, University of Cambridge, Cambridge, UK; 4 Neurosciences and Trauma Critical Care Unit, Addenbrooke’s Hospital, Cambridge University Hospitals, Cambridge, UK

**Keywords:** Cerebral perfusion pressure, Intracranial pressure, Pressure reactivity index, Traumatic brain injury, Brain tissue oxygenation, Multimodality monitoring

## Abstract

**Background:**

The primary aim was to explore the association of global cerebral physiological variables including intracranial pressure (ICP), cerebrovascular reactivity (PRx), cerebral perfusion pressure (CPP), and deviation from the PRx-based optimal CPP value (∆CPPopt; actual CPP-CPPopt) in relation to brain tissue oxygenation (pbtO_2_) in traumatic brain injury (TBI).

**Methods:**

A total of 425 TBI patients with ICP- and pbtO_2_ monitoring for at least 12 h, who had been treated at the neurocritical care unit, Addenbrooke’s Hospital, Cambridge, UK, between 2002 and 2022 were included. Generalized additive models (GAMs) and linear mixed effect models were used to explore the association of ICP, PRx, CPP, and CPPopt in relation to pbtO_2_. PbtO_2_ < 20 mmHg, ICP > 20 mmHg, PRx > 0.30, CPP < 60 mmHg, and ∆CPPopt < − 5 mmHg were considered as cerebral insults.

**Results:**

PbtO_2_ < 20 mmHg occurred in median during 17% of the monitoring time and in less than 5% in combination with ICP > 20 mmHg, PRx > 0.30, CPP < 60 mmHg, or ∆CPPopt < − 5 mmHg. In GAM analyses, pbtO_2_ remained around 25 mmHg over a large range of ICP ([0;50] mmHg) and PRx [− 1;1], but deteriorated below 20 mmHg for extremely low CPP below 30 mmHg and ∆CPPopt below − 30 mmHg. In linear mixed effect models, ICP, CPP, PRx, and ∆CPPopt were significantly associated with pbtO_2_, but the fixed effects could only explain a very small extent of the pbtO_2_ variation.

**Conclusions:**

PbtO_2_ below 20 mmHg was relatively frequent and often occurred in the absence of disturbances in ICP, PRx, CPP, and ∆CPPopt. There were significant, but weak associations between the global cerebral physiological variables and pbtO_2_, suggesting that hypoxic pbtO_2_ is often a complex and independent pathophysiological event. Thus, other variables may be more crucial to explain pbtO_2_ and, likewise, pbtO_2_ may not be a suitable outcome measure to determine whether global cerebral blood flow optimization such as CPPopt therapy is successful.

**Supplementary Information:**

The online version contains supplementary material available at 10.1186/s13054-023-04627-y.

## Introduction

Traditional neurocritical care (NCC) for patients with traumatic brain injury (TBI) has focused on monitoring and treatment of intracranial pressure (ICP) and cerebral perfusion pressure (CPP) to avoid brain herniation and ischaemia [1–4]. However, it has been realized that these patients also exhibit disrupted cerebral pressure autoregulation, oxygen diffusion limitations, and mitochondrial dysfunction that contribute to energy failure and secondary brain injury [1, 5–9]. Current research aims at utilizing multimodality monitoring techniques and advanced software to diagnose these pathophysiological events in order to better guide clinical management [10].

A leading approach to detect cerebral pressure autoregulatory disturbances is based on the pressure reactivity index (PRx), which is assessed as the moving correlation coefficient between slow changes in arterial blood pressure (ABP) and ICP. Negative and positive PRx-values indicate preserved and impaired autoregulation, respectively [11]. PRx often varies with CPP in a U-shaped way over a certain buffer of time, and the CPP with the lowest PRx has been denoted as the optimal CPP (CPPopt) [12, 13]. CPPopt differs between TBI patients and changes over time for the individual patient [12–15]. CPPopt is hypothesized to be a better CBF surrogate than fixed, absolute CPP targets [16, 17]. Consistently, in observational TBI studies, CPP close to CPPopt has been associated with better brain tissue oxygenation (pbtO_2_) [18, 19], energy metabolism [20], and favourable outcome [12–14, 21, 22]. A phase II-trial recently demonstrated that CPPopt-guided therapy is safe and feasible in TBI [15], but a phase III-trial is needed to determine whether targeting CPPopt translates into improved cerebral physiology and functional outcome.

PbtO_2_, which is measured with a focal probe in the brain, is a downstream variable to CBF that has received great interest as a potential treatment target in TBI [23]. Low pbtO_2_ is associated with unfavourable outcome [24–28] and a phase II-trial recently demonstrated that integrated management based on pbtO_2_ together with ICP was safe, feasible, and with a trend towards lower mortality compared with ICP-management alone [29]. There are now three ongoing randomized controlled trials (BOOST-3, OXY-TC, and BONANZA) aimed at evaluating if pbtO_2_-directed therapy adds any clinical value in terms of outcomes [23]. Current pbtO_2_-integrated protocols are tailored based on the combination of ICP-, CPP-, and pbtO_2_-insults. In cases with low pbtO_2_ in the absence of high ICP and low CPP, therapy is aimed at increasing cerebral oxygen delivery by augmentation of CBF and the arterial oxygen content [23, 30]. Interestingly, although cerebral ischaemia is one reason for brain tissue hypoxia in TBI, retrospective studies indicate that low pbtO_2_ typically occurs despite well-targeted global cerebral physiological variables (ICP and CPP) [27, 31]. One explanation could be that absolute CPP is a poor CBF surrogate and deviation from CPPopt towards lower CPP might be more sensitive to ischaemic hypoxia [18, 19, 32]. Another explanation is that cerebral ischaemia and hypoxia are spatially distributed throughout the brain and only overlap to some extent after TBI [33]. Thus, global CBF and its descriptors may be poor surrogates of focal brain tissue hypoxia. Instead, capillary flow disturbances and oxygen diffusion limitations may explain the impeded oxygen delivery in these cases of low pbtO_2_ in the absence of focal ischaemia [6–8].

Altogether, CPPopt may be a better global CBF surrogate than absolute CPP and could be a means to fine-tune CBF optimization, particularly in case of hypoxic values of pbtO_2_. At the same time, pbtO_2_ is a downstream variable to CBF and could be used as a target to determine whether CPPopt therapy is successful and within which range CPPopt should be targeted. However, the relative contribution of CPP/CPPopt/CBF to pbtO_2_ may be limited as low pbtO_2_ may often reflect non-perfusion-related disturbances in TBI. To better understand these uncertainties, we performed a two-part study on the role of pbtO_2_ monitoring in TBI. Here we present part I of our study, where we aimed to explore the influence of ICP, PRx, CPP, and deviation from CPPopt on pbtO_2_ in a large cohort of TBI patients with pbtO_2_ and high-frequency ICP and ABP monitoring data. Our hypothesis was that the global cerebral physiological variables described above would be associated with pbtO_2_, but only explain a small part of its variations. In part II, we explored the prognostic value of pbtO_2_ in TBI patients, and this will be presented in a separate manuscript.

## Materials and methods

### Patients and study design

In this observational, single-centre study, 781 de-identified records of high-resolution neuromonitoring data of TBI patients admitted in the NCC unit of the Addenbrooke’s Hospital (Cambridge, UK) between 2002 and 2022, were screened from the Brain Physics Lab research database. All patients had ABP and ICP data, 461 of these patients were also monitored with pbtO_2_, of which 36 records had less than 12 h of data and these were excluded. Thus, the final population included those 425 TBI patients with ICP, ABP, and pbtO_2_ data for more than 12 h, and they were admitted between 2006 and 2022. Ten-second averages of pre-processed cleaned ABP, ICP, and pbtO_2_ data were accessed. The following clinical descriptors were also retrieved: age, sex, Glasgow Coma Scale (GCS), pupillary reactivity, decompressive craniectomy, and Glasgow outcome scale (GOS).

### Management protocol

The management protocol has been described in detail in previous studies [2, 34]. The protocol went through slight revisions during the study period of 20 years [2]. CPP was targeted above 60 mmHg, ICP below 20 and 22 mmHg, pbtO_2_ above 20 mmHg, partial pressure of carbon dioxide within 4.5–5 kPa, and arterial glucose within 6–8 mmol/L. PRx and CPPopt were introduced at the bedside in 1999 and 2012, respectively, and were primarily used as monitoring variables. However, CPPopt could also be targeted at the discretion of the responsible clinician. Unconscious patients (GCS < 9) were intubated, mechanically ventilated, and sedated. ICP-treatments included head elevation, sedation, muscle paralysis, cerebrospinal fluid drainage via an external ventricular drainage, osmotic agents, induced hypothermia, barbiturate coma, and decompressive craniectomy. CPP was maintained at first hand by intravenous fluids and at second hand with vasopressors/inotropes. PbtO_2_ was mainly maintained above 20 mmHg by reducing ICP and increasing CPP. Additional efforts to keep pbtO_2_ above the designated target were allowed based on the treating physician's discretion. This could involve raising the fraction of inspired oxygen (FiO_2_) levels or implementing a moderate upward adjustment of CPP.

### Clinical outcome

Clinical outcome was evaluated according to the GOS at 6 months post-injury, by clinical assessment or via telephone interviews by trained staff. GOS ranges from 1 (death) to 5 (good recovery) [35, 36]. Outcome was dichotomized as favourable and unfavourable outcome (GOS 4–5 and 1–3, respectively) as well as survival and mortality (GOS 2–5 and 1, respectively).

### Physiological measurements

ICP (Codman ICP MicroSensor, Codman & Shurtleff, Raynham, Massachusetts) and pbtO_2_ (Licox, Integra Neurosciences, Andover, UK), were monitored with intraparenchymal probes in the non-dominant frontal lobe. Direct placement of the pbtO_2_ probe into lesions was avoided. ICP could also be monitored by external ventricular drain (EVD), ABP was monitored in the radial or femoral artery (Baxter Healthcare, Deerfield, Illinois) with a zero calibration at the level of the right atrium (2002–2015) and at the foramen of Monroe (2015–2022). Physiological data were streamed in real time from the monitors into the ICM + software running on a laptop at the bedside (ICM + software, Cambridge Enterprises, University of Cambridge, UK) [https://icmplus.neurosurg.cam.ac.uk], which provided data integration and storage at sampling rate from 50 to 250 Hz, and de-identification for the Brain Physics Lab research database.

#### Data processing

ICM+ software was used for data pre-processing prior to storage into the database according to local protocols, briefly outlined here. The data were first curated manually for large artefacts. Further, automated artefact mark-up was applied. PbtO_2_ values below 5 mmHg or above 100 mmHg were rejected. ABP artefacts were removed automatically in case of absence of detected pulse, or when diastolic values fell to below a threshold of 15 mmHg. Automatic ICP artefacts removal used low amplitude values (< 0.04 mmHg) to reject artefactual periods, which corresponded to either noise or short recording periods during which EVD was kept open (for those recordings where ICP was measured via EVD). 95% Spectral edge frequency above 10 Hz was used to reject periods of non-physiological measurements of ICP. All recorded signals were subsequently down-sampled to 0.1 Hz by coarse graining using 10 s, non-overlapping averages.

The 10-s physiological data were accessed from the database and were further manually scrutinized and cleaned of large artefacts for the scope of this study. PRx was calculated as the moving Pearson correlation coefficient of 30 consecutive 10-s average values of ABP and ICP and updated every minute [11, 37]. Minute-by-minute values of CPPopt were calculated as the CPP with the concurrently lowest PRx, using the multi-window weighted algorithm based on a data buffer of 2 to 8 h [38]. ∆CPPopt was defined as the difference between actual CPP and CPPopt.

#### Measurements

The physiological variables were analysed over the entire NCC monitoring period. The percentage of good monitoring time (GMT, i.e. the remaining coverage of data after cleaning as described above) with pbtO_2_ < 20 mmHg, ICP > 20 and 22 mmHg, PRx > 0.00 and 0.30, CPP < 60 mmHg, and ∆CPPopt < -5 and − 10 mmHg, were analysed. In addition, the combination of pbtO_2_ < 20 mmHg together with ICP > 20/22 mmHg, PRx > 0.00/0.30, CPP < 60 mmHg, or ∆CPPopt < − 5/− 10 mmHg, respectively, of the GMT were calculated. The pbtO_2_-threshold at 20 mmHg was chosen in accordance with the current randomized controlled trials [23]. The thresholds of ICP at 20 and 22 mmHg and CPP at 60 mmHg were chosen in accordance with our management protocol and international guidelines [39]. This PRx-threshold at 0.00 indicates the transition from negative to positive ABP/ICP correlation, while a threshold close to 0.30 has previously been strongly associated with unfavourable outcome [40] and been suggested as the approximate threshold for identifying limits of autoregulation [41]. ∆CPPopt < − 5 mmHg was previously suggested as the autoregulatory threshold for hypoperfusion [12], while ∆CPPopt < − 10 mmHg was considered a severe insult.

The data were summarized both as minute-by-minute and hourly values for the statistical analyses. The hourly values were also sub-classified into sequenced blocks for every coherent period of pbtO_2_ monitoring without a data gap. For the hourly data, separate variables of negative/positive ∆CPPopt below/above 0 were calculated. Every minute-value of ∆CPPopt > 0 was counted as 0 and every ∆CPPopt < 0 was counted as a positive mmHg (e.g. CPP at 75 mmHg and CPPopt at 80 mmHg yielded a positive value at 5 mmHg) when hourly values of negative ∆CPPopt were calculated. Similarly, every minute-value of ∆CPPopt < 0 was counted as 0 and every ∆CPPopt > 0 was counted as a positive mmHg when hourly values of positive ∆CPPopt was calculated. This was done to reduce the risk that ∆CPPopt was averaged to 0 in patients with high blood pressure variability with both hypo- and hyperperfusion [42].

### Statistical analysis

The statistical analyses were conducted in RStudio software (version 2022.12.0) [43]. Descriptive data of demographics, treatment variables, clinical outcome, and physiological variables were described as number (proportion) and median (interquartile range (IQR)). The association between pbtO_2_ in relation to ICP, PRx, CPP, and ∆CPPopt, respectively, was analysed with generalized additive models (GAMs) with cubic spline and according to the restricted maximum likelihood, based on minute-by-minute data. This approach allowed us to explore large amounts of data points and to capture the shape of the relationship between two variables without choosing a particular parametric form a priori. This means that we could create a regression line that allowed to reveal nonlinear associations between the variables. Sub-analyses of these GAMs for ICP, CPP, and ∆CPPopt in relation to pbtO_2_ were conducted based on filtered data when autoregulation was intact or impaired autoregulation (PRx below or above 0.30). Similar GAMs were computed for the subgroup of patients who were not treated with decompressive craniectomy.

Linear mixed effect models (R package lme4) based on hourly monitoring data with pbtO_2_ as the dependent variable and the global physiology-related variables as the independent variables were also done. This approach allowed us to adjust for potential patient-specific and temporal effects. Two separate models were used. One was based on absolute CPP-values and included ICP, PRx (above/below 0.30), CPP, the interaction between ICP and CPP with PRx, and time from start of monitoring as fixed effects and patients, time from monitoring, and the specific monitoring sequence as random effects. Stepwise backward model reduction was done to exclude variables that were not significant (*p* > 0.05). A similar model was done with negative/positive ∆CPPopt instead of CPP as an independent variable. Model assumptions were evaluated and data transformation was applied as appropriate to meet the linearity assumption. Conditional and marginal R squared (R package performance v 0.1.0.0) were assessed in accordance with Nakagawa et al. [44]. A *p*-value below 0.05 was considered statistically significant.

## Results

### Demography, clinical variables, treatments, and outcome

In this study with 425 TBI patients (Table 1), median age was 35 (25–55) years and the male/female ratio was 260/65 (80/20%). At presentation, GCS was in median 7 (IQR 4–10) and pupillary reactivity was preserved bilaterally in 150 (84%), unilaterally unreactive in 20 (11%), and bilaterally unreactive in 8 (4%) cases. Seventy-three (25%) patients were treated with decompressive craniectomy. At 6 months, median GOS was 4 (IQR 2–4), 124/115 (52/48%) recovered favourably/unfavourably, and 58/181(24/76%) had deceased/survived. Clinical and outcome data were unavailable in some cases, as outlined in the table legend.

**Table 1:** Demographics, admission variables, treatments, and clinical outcome

Patients, *n* (%)	425 (100%)
Age (years), median (IQR)	35 (25–55)
Sex (male/female), *n* (%)	260/65 (80/20%)
GCS, median (IQR)	7 (4–10)
Pupillary reactivity (intact/unilaterally unreactive/bilaterally unreactive), *n* (%)	150/20/8 (84.3/11.2/4.5%)
Decompressive craniectomy, *n* (%)	73 (25%)
GOS, median (IQR)	4 (2–4)
Favourable/unfavourable outcome, i (%)	124/115 (52/48%)
Mortality, *n* (%)	58 (24%)

Missing data: age (*n* = 62), sex (*n* = 100), GCS (*n* = 139), pupillary reactivity (*n* = 247), decompressive craniectomy (*n* = 130), GOS (*n* = 186)

*GCS* Glasgow comas scale, *GOS* Glasgow outcome scale, *IQR* interquartile range

### Cerebral physiological variables and secondary insults during neurocritical care

The median values of the cerebral physiological variables during NCC are described in Table 2. ICP insults above 20 mmHg occurred during 5 (IQR 2–20) % of the GMT, PRx-insults above 0.30 occurred during 23 (IQR 15–35) % of the GMT, CPP-insults below 60 mmHg occurred during 3 (IRQ 1–7) % of GMT, ∆CPPopt below − 5 mmHg during 28 (IQR 20–36) % of GMT, and pbtO_2_ < 20 mmHg during 17 (IQR 2–49) % of GMT. PbtO_2_ below 20 mmHg in combination with either ICP above 20/22 mmHg, PRx above 0.30, CPP below 60 mmHg, or ∆CPPopt below − 5/− 10 mmHg occurred in less than 5% of GMT. The number of days with available data of ICP and pbtO_2_ was in median 6 (IQR 3–10) and 4 (IQR 2–5), respectively.

**Table 2:** Physiological variables and burden of secondary insults during neurocritical care

*Physiological variables*	
ICP (mmHg), median (IQR)	12 (8–16)
PRx (coefficient), median (IQR)	0.01 (− 0.11–0.14)
CPP (mmHg), median (IQR)	75 (72–79)
CPPopt (mmHg), median (IQR)	75 (71–80)
PbtO_2_ (mmHg), median (IQR)	26 (20–28)
*Insults*	
ICP > 20 mmHg (%GMT), median (IQR)	5 (2–20)
ICP > 22 mmHg (%GMT), median (IQR)	3 (1–11)
PRx > 0.00 (%GMT), median (IQR)	51 (38–64)
PRx > 0.30 (%GMT), median (IQR)	23 (15–35)
CPP < 60 mmHg (%GMT), median (IQR)	3 (1–7)
∆CPPopt < − 5 mmHg (%GMT), median (IQR)	28 (20–36)
∆CPPopt < −10 mmHg (%GMT), median (IQR)	14 (8–20)
PbtO_2_ < 20 mmHg (%GMT), median (IQR)	17 (2–49)
*Combined pbtO*_*2*_* and global cerebral physiology-insults*	
PbtO_2_ < 20 mmHg and ICP > 20 mmHg for (%GMT), median (IQR)	1 (0–3)
PbtO_2_ < 20 mmHg and ICP > 22 mmHg for (%GMT), median (IQR)	0 (0–2)
PbtO_2_ < 20 mmHg and PRx > 0.00 (%GMT), median (IQR)	6 (1–21)
PbtO_2_ < 20 mmHg and PRx > 0.30 (%GMT), median (IQR)	1 (0–6)
PbtO_2_ < 20 mmHg and CPP < 60 mmHg (%GMT), median (IQR)	0 (0–1)
PbtO_2_ < 20 mmHg and ∆CPPopt < − 5 mmHg (%GMT), median (IQR)	4 (0–11)
PbtO_2_ < 20 mmHg and ∆CPPopt < − 10 mmHg (%GMT), median (IQR)	3 (0–9)

*CPP* cerebral perfusion pressure, *CPPopt* optimal CPP, *GMT* good monitoring time, *ICP* intracranial pressure, *IQR* interquartile range, *PbtO*_*2*_ partial brain tissue oxygenation, *PRx* pressure reactivity index

### PbtO_2_ in relation to global cerebral physiology-related variables

**Fig. 1:**
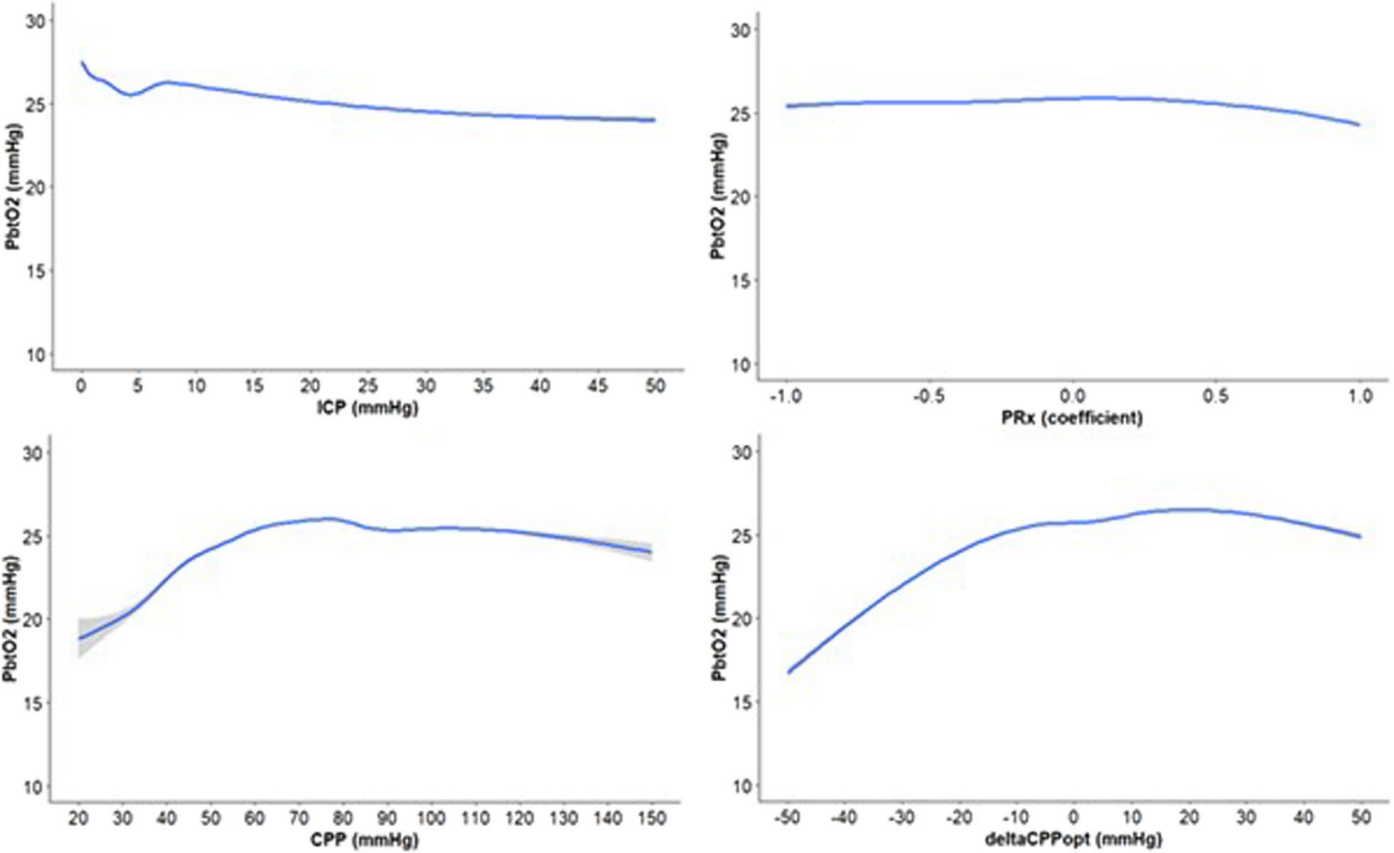
PbtO_2_ in relation to cerebral physiological variables—a GAM analysis. In these GAM analyses, pbtO_2_ remained within 20–30 mmHg over the range of ICP between 0 to 50 mmHg and of PRx between − 1 and + 1. PbtO_2_ plateaued at 25 mmHg for CPP above 60–70 mmHg, gradually decreased for lower CPP-values, and even went below 20 mmHg when CPP was below 30 mmHg. Similarly, pbtO_2_ plateaued around 25 mmHg for ∆CPPopt of − 10 mmHg and higher, but went below 20 mmHg for ∆CPPopt below − 30 mmHg. *CPP* cerebral perfusion pressure, *GAM* generalized additive model, *ICP* intracranial pressure, *PbtO*_2_ partial brain tissue oxygenation, *PRx* pressure reactivity index

**Fig. 2:**
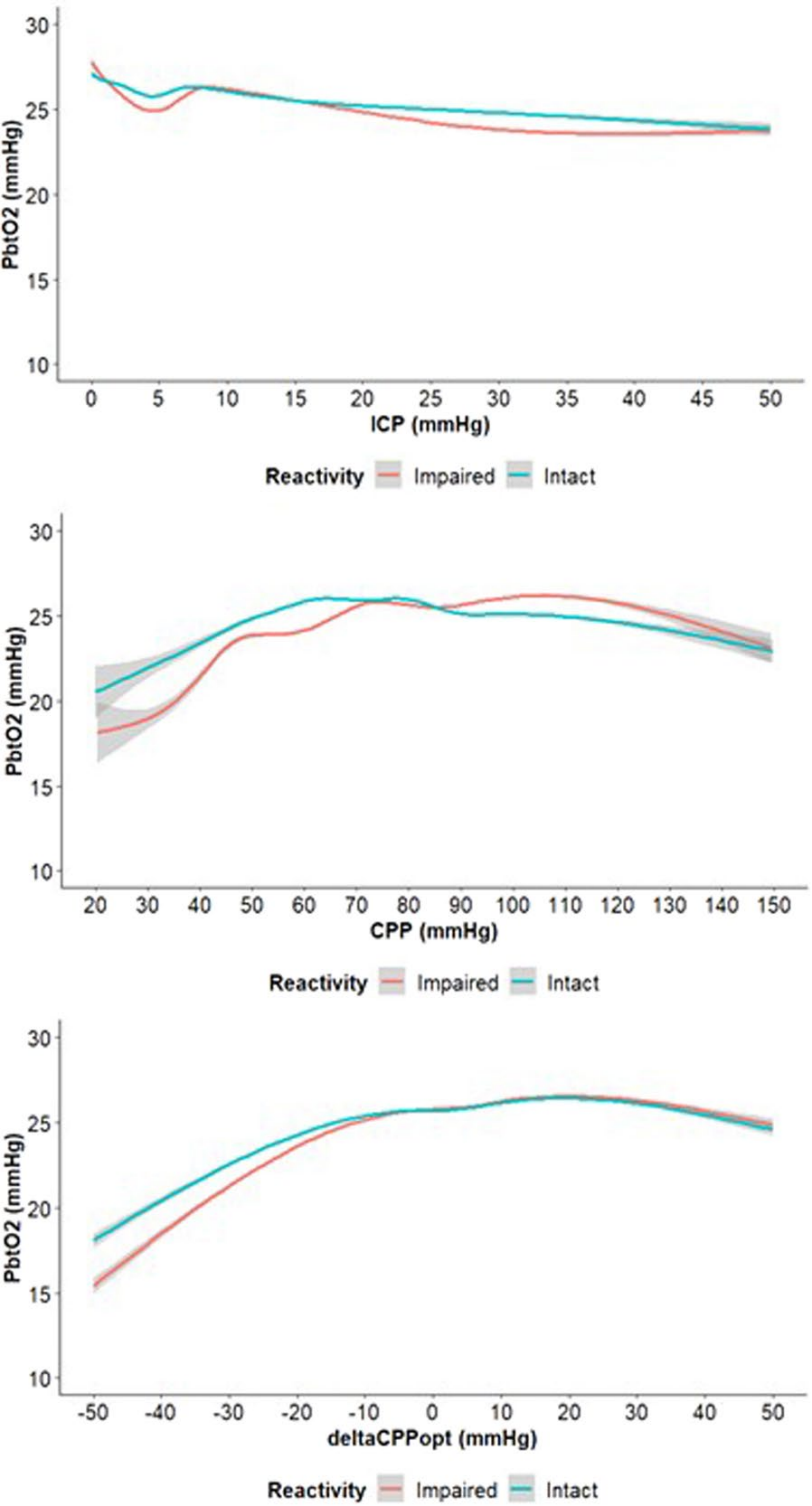
PbtO_2_ in relation to cerebral physiological variables depending on the concurrent pressure or oxygen reactivity status—a GAM analysis. Impaired/intact reactivity was defined as PRx above/below 0.30. As illustrated, pbtO_2_ was generally minimally lower over the ICP-range when pressure autoregulation was impaired. In addition, pbtO_2_ was slightly lower for CPP below 60–70 mmHg when pressure autoregulation was impaired, but minimally higher for higher CPP-values. Similarly, pbtO_2_ was slightly lower for negative ∆CPPopt when pressure autoregulation was impaired, but minimally higher for positive ∆CPPopt. *CPP* cerebral perfusion pressure. *GAM* generalized additive model, *ICP* intracranial pressure, *PbtO*_*2*_ partial brain tissue oxygenation, *PRx* pressure reactivity index

In GAM analyses, pbtO_2_ remained within 20 to 30 mmHg over the range of ICP between 0 to 50 mmHg and PRx between − 1 and + 1 (Fig. 1). PbtO_2_ plateaued at 25 mmHg for CPP above 60–70 mmHg, gradually decreased for lower CPP-values, and even went below 20 mmHg when CPP was below 30 mmHg. Similarly, pbtO_2_ plateaued at 25 mmHg around ∆CPPopt of − 10 mmHg and higher, but went below 20 mmHg for ∆CPPopt below − 30 mmHg. Furthermore, GAMs (Fig. 2) with regression lines separated depending on if they were based on minute-values when pressure autoregulation was intact or impaired (PRx below or above 0.30) were conducted. As illustrated, pbtO_2_ was generally minimally lower over the ICP-range when pressure autoregulation was impaired. In addition, pbtO_2_ was slightly lower for CPP below 60–70 mmHg when pressure autoregulation was impaired, but minimally higher for higher CPP-values. Similarly, pbtO_2_ was slightly lower for negative ∆CPPopt when pressure autoregulation was impaired, but minimally higher for positive ∆CPPopt. Similar GAMs were done for patients who had not undergone decompressive craniectomy (Additional files: 1 and 2), which showed slight differences in the regression lines at the extreme values of the global cerebral physiological variables as compared to the entire cohort.

In a linear mixed effect model based on absolute CPP (Table 3), higher ICP, intact pressure autoregulation, and higher CPP were associated with higher pbtO_2_. The association between CPP and pbtO_2_ was attenuated if pressure autoregulation was intact. In a similar linear mixed model where ∆CPPopt had replaced absolute CPP, higher ICP, impaired autoregulation, and absence of negative ∆CPPopt were associated with higher pbtO_2_. The association between negative ∆CPPopt and pbtO_2_ was attenuated if pressure autoregulation was intact. Conditional squared and marginal R squared suggested that only 3.5% (CPP model) and 1.5% (CPPopt model) of the variance was explained by the fixed effects, while majority of the variance was explained by the random effects.

**Table 3:** Global physiology-related variables in relation to pbtO_2_—linear mixed effect model analyses

Variable	Value	SE	*p*
*CPP model*			
Intercept	19.19	0.81	< 0.001
ICP (mmHg)	0.16	0.01	< 0.001
Intact autoregulation (yes)*	1.70	0.49	< 0.001
CPP (mmHg)	0.10	0.01	< 0.001
CPP if intact autoregulation (yes)**	− 0.02	0.01	< 0.001
*CPPopt model*			
Intercept	27.83	0.63	< 0.001
ICP (mmHg)	0.12	0.01	< 0.001
Intact autoregulation (yes)*	− 0.73	0.11	< 0.001
Negative ∆CPPopt (mmHg)	− 0.14	0.01	< 0.001
Negative ∆CPPopt if intact autoregulation (yes)**	0.07	0.01	< 0.001

Two separate models were used to predict hourly values of pbtO_2_ based on the global perfusion-related variables, one with absolute CPP and another with ∆CPPopt (mean hourly positive ∆CPPopt > 0 and negative ∆CPPopt < 0). The model based on absolute CPP-values also included ICP, PRx (above/below 0.30), and the interaction between ICP and CPP with PRx, and time from start of monitoring as fixed effects. Random effects were time from monitoring (slope) and patients and sequence (nested intercepts). Stepwise backward model reduction was done to exclude fixed effects variables that were not significant (*p* > 0.05) and the table shows the final model after these procedures. AIC was 327,026, conditional R squared was 0.467 and marginal R squared was 0.035. When the random slope (time) was added to the fixed effects for appropriate assessment of the conditional random effect variance, the conditional R squared was 0.996 and the marginal R squared was 0. A similar model was done with negative/positive ∆CPPopt instead of CPP as an independent variable and the table shows the final model after these procedures. Negative ∆CPPopt was reported as an absolute value, i.e. a higher negative ∆CPPopt was associated with a lower pbtO_2_ in the model above. Positive ∆CPPopt was not significant and therefore removed from the model. AIC was 284,519, conditional R squared was 0.457 and marginal R squared was 0.015. When the random slope (time) was added to the fixed effects for appropriate assessment of the conditional random effect variance, the conditional R squared was 0.996 and the marginal R squared was 0. The models were explored with and without standardization of the data, achieving the same results.”

*AIC* akaike information criterion, *CPP* cerebral perfusion pressure, *CPPopt* optimal CPP, *ICP* intracranial pressure, *PbtO*_*2*_ partial brain tissue oxygenation, *PRx* pressure reactivity index, *SE* Standard error

*Intact autoregulation; PRx < 0.30

**Interactions

## Discussion

In this observational study including 425 TBI patients, the main findings were that low pbtO_2_ was fairly common and these events often occurred in the absence of disturbances in ICP, PRx, CPP, and ∆CPPopt. Although these global cerebral physiological variables were indeed associated with pbtO_2_ in linear mixed effect models, even large changes in these variables only had a modest effect on pbtO_2_. Otherwise, ICP and CPP were typically controlled and such insults were rare during NCC. This could explain their limited association with low pbtO_2_, whereas the relative contribution of non-perfusion-related pathophysiological mechanisms such as hypoxemia, capillary dysfunction, and oxygen diffusion limitations might have had a greater impact on pbtO_2_. The weak associations between the global cerebral physiological variables and pbtO_2_ implies that other parameters may be more crucial to affect pbtO_2_. It also indicates that pbtO_2_ may not be a suitable outcome measure to determine whether global cerebral blood flow optimization such as CPPopt therapy is successful.

First, we found that pbtO_2_-insults below 20 mmHg were relatively common and occurred, in median, during 17% of the GMT. Interestingly, ICP-, PRx-, CPP-, and ∆CPPopt-insults were relatively infrequent during these periods of low pbtO_2_, indicating that the latter was often an independent pathophysiological event. This observation corroborates findings in previous smaller studies [27, 31].

Second, when pbtO_2_ was explored in relation to ICP, PRx, CPP, and ∆CPPopt, we found variability in pbtO_2_ to be weakly associated with these variables in the GAMs and linear mixed effect models. However, pbtO_2_ essentially remained stable within large ranges of these variables. A larger pbtO_2_ deterioration with at least 5 mmHg to values below 20 mmHg was chiefly seen at extremely low values of CPP and ∆CPPopt, i.e. when the entire brain was likely to be affected by ischaemic hypoxia. The GAMs showed that pbtO_2_ increased modestly between ICP 5–10 mmHg and thereafter slightly declined, whereas higher ICP was associated with small increases in pbtO_2_ in the linear mixed models. This discrepancy could be explained by the limitations of the linear assumptions in the linear mixed model. However, the main message from both the GAM and linear mixed models is still that great changes in the global cerebral physiological variables only led to small changes in pbtO_2_.

Third, PRx influenced the associations of particularly CPP/∆CPPopt and pbtO_2_, but, again, only with small magnitudes. Particularly, impaired pressure reactivity increased the susceptibility for ischaemic hypoxia when CPP went below 60–70 mmHg. This was expected, since impaired pressure reactivity at the lower limit of autoregulation implies a reduced capacity to maintain CBF and brain tissue oxygenation. Although CBF was expected to remain stable even for very low CPP when pressure autoregulation was intact thanks to compensatory vasodilation, we did see slight deteriorations in pbtO_2_ for very low CPP even when PRx was low. This challenges the traditional view of pressure autoregulation to some extent, but is consistent with a recent experimental pig study, which demonstrated that CBF starts to deteriorate slightly before the entire vasodilatory reserve/pressure autoregulation is exhausted [45]. Thus, cerebral ischaemia and hypoxia could still occur for low CPP even when PRx is relatively low. For higher CPP-values, pbtO_2_ remained relatively constant with a tendency towards slightly lower values as CPP reached above 100 mmHg. This was in contrast to a smaller, exploratory TBI study showing that pbtO_2_ continues to increase when CPP reaches very high levels, consistent with the idea that higher CPP yields higher CBF, oxygen delivery, and ultimately pbtO_2_ [19]. Although this makes sense, there are several general physiological as well as TBI pathophysiological mechanisms that may alter the association between high CPP and pbtO_2_. Particularly, even when CBF starts to increase after CPP has exceeded the upper breakpoint of autoregulation, pbtO_2_ will only increase to some extent until it is saturated as the vascular and tissue oxygen gradient gradually decreases [8, 46]. In addition, TBI typically leads to capillary dysfunction due to astrocyte swelling and microvascular thrombosis, which increases capillary transit time heterogeneity and to various degrees impedes focal oxygen delivery and extraction [8, 46]. The extent of capillary flow disturbances modulates the effect of CPP and CBF on pbtO_2_ and higher focal CBF may even decrease oxygen delivery when the capillaries are severely injured [8, 46]. Furthermore, high CPP may increase brain oedema, which could increase oxygen diffusion limitations and thereby decrease pbtO_2_ [30, 47]. Thus, many factors may contribute to the relation between CPP/CBF in the higher range and pbtO_2_ [48]. Lastly, for the autoregulatory perfusion variable ∆CPPopt, pbtO_2_ deteriorated when ∆CPPopt went to very negative values below − 20 to − 30 mmHg, but pbtO_2_ was otherwise relatively stable at 25 mmHg for ∆CPPopt above such low values. PbtO_2_ was slightly lower for any given negative ∆CPPopt when pressure autoregulation was impaired (high PRx). Again, this supports the experimental findings that ischaemia may begin while the cerebral vessels are to some extent still reactive [45]. This also highlights the importance of the CPPopt curve shape and the role of absolute PRx-values in CPPopt therapy [21].

Altogether, pbtO_2_ was primarily sensitive to global cerebral physiological variables when the latter were severely deranged, i.e. when the entire brain was affected by severe ischaemic hypoxia, but otherwise to a very limited extent. This was expected for several reasons. Within individual TBI patients, there is a great spatial CBF heterogeneity as ischaemia and hyperaemia can co-exist in different brain regions at the same time [48]. In addition, brain tissue hypoxia is also usually spatially distributed in the brain for individual TBI patients, but only overlaps with ischaemic areas to some extent [33]. In addition, pbtO_2_ is a sum measure of focal CBF, arterial oxygen content, oxygen diffusion, energy requirement (seizures, sedation), and mitochondrial function (capacity for oxygen utilization), so global cerebral physiological variables may only explain variations in pbtO_2_ to some degree [5, 33]. Since ICP, PRx, CPP, and ∆CPPopt are all global variables, whereas pbtO_2_ is focally limited to a small brain area it would be expected to mainly correlate with the global variables when the entire brain is affected.

### Methodological considerations

The main strength of this manuscript was the large cohort size (*n* = 425) of TBI patients with high-frequency data of ICP and pbtO_2_. We also applied advanced statistical approaches to explore potential nonlinear associations (GAM analyses) as well as time- and patient-related factors (linear mixed effect models) in the physiological analyses of global cerebral physiological variables in relation to pbtO_2_.

There were also some limitations. First, demographic, clinical, imaging, and outcome data could not be retrieved in some cases. Particularly, this inhibited us from proceeding with classifications based on pbtO_2_ probe location (peri-lesional and normal-appearing brain). The proximity for the pbtO_2_ probe to any focal lesion might have affected the association between cerebral physiology and pbtO_2_ [26, 49]. However, in the clinical management, placement of the pbtO_2_ probes close to lesions was avoided. We also addressed the role of the probe location in the linear mixed effect models by taking into account patients (and inherently their probe location) as a random factor. Still, this could not be done in the GAMs and more detailed data of the probe locations would have made our analyses more granular and clearer. Third, the reliability of PRx and thus CPPopt post-craniectomy has been questioned. Preliminary studies indicate that the reliability of these variables may be preserved after craniectomy [50] and we therefore decided to keep these patients in the main analyses, but also explored excluding them in the GAMs. The reliability of PRx and CPPopt has also been questioned for EVD based ICP measurements, particularly if the protocol management requires regular opening and closing of the EVD. Since we could not clearly identify all the patients that had EVD, we attempted to address this issue with careful signal processing. Fourth, this was a single-centre study with a selection of those patients who received both ICP- and pbtO_2_ monitoring. This limits the external validity of our findings. Fifth, in addition to perfusion-related variables and probe location, many other variables that were not investigated here might have influenced pbtO_2_, such as arterial oxygen content (haemoglobin and pO_2_) and cerebral energy metabolism (temperature, seizures). All of these were also to various degrees influenced by a complex NCC management including e.g. red blood cell transfusions, ventilatory settings, fever management, and sedation. The relative importance of these variables on pbtO_2_ should be further explored in future studies. In addition, the patients were treated and optimized at an NCC, particularly according to an ICP-/CPP-oriented protocol. It is possible that the association between the global cerebral physiological variables and pbtO_2_ would have been different if the patients had been treated according to another regime or if the variables had been less strictly optimized. Most likely, the attentive NCC management attenuated the association between the global cerebral physiological variables and pbtO_2_ in our analyses (GAMs and linear mixed effect models).

## Conclusions

PbtO_2_ below 20 mmHg was relatively common after moderate to severe TBI. This typically occurred despite adequate ICP, PRx, CPP, and ∆CPPopt. Although these global cerebral physiological variables were associated with pbtO_2_, this only explained a small part of the variation in brain tissue oxygenation. This could be viewed in light of previous studies showing great spatial CBF heterogeneity between brain regions within patients and limited overlap between focal ischaemia and hypoxia. This is of great importance since ICP, PRx, CPP, and ∆CPPopt are global cerebral physiological variables, whereas pbtO_2_ is measured focally. Considering the weak associations between perfusion-related variables and pbtO_2_, our findings suggest that pbtO_2_ may overall be a poor indicator of global CBF homeostasis, except for extremely low CPP/negative ∆CPPopt when the entire brain is affected. Consequently, pbtO_2_ might not be a suitable outcome measure to determine whether global CBF optimization such as CPPopt-guided therapy is successful, although it may convey other pathophysiological information such as focal disturbances in capillary blood flow and oxygen diffusion limitations. To understand the potential clinical value of pbtO_2_ better, the prognostic role of exhibiting pbtO_2_ below certain critical thresholds was investigated in the part II study.

### Supplementary Information

**Additional file 1.** PbtO_2_ in relation to cerebral physiological variables for patients who were not treated with decompressive craniectomy – a GAM analysis.

**Additional file 2.** PbtO_2_ in relation to cerebral physiological variables depending on the concurrent pressure or oxygen reactivity status for patients who were not treated with decompressive craniectomy – a GAM analysis.

## Data Availability

Data are available upon reasonable request.

## Abbreviations

ABP: Arterial blood pressure
AIC: Akaike information criterion
CBF: Cerebral blood flow
CPP: Cerebral perfusion pressure
CPPopt: Optimal CPP
GAM: Generalized additive model
GMT: Good monitoring time
GOS: Glasgow outcome scale
ICP: Intracranial pressure
IQR: Interquartile range
NCC: Neurocritical care
PbtO_2_: Partial brain tissue oxygenation
PRx: Pressure reactivity index
SE: Standard error
TBI: Traumatic brain injury
