# Supplementary material for: Brain tissue oxygen monitoring in traumatic brain injury: part I—To what extent does PbtO2 reflect global cerebral physiology?
Source: Crit Care. 2023 Aug 31;27:339. doi: 10.1186/s13054-023-04627-y (PMC10472704; doi:10.1186/s13054-023-04627-y)
Supplement: Supplementary file 2 — Additional file 2. PbtO2 in relation to cerebral physiological variables depending on the concurrent pressure or oxygen reactivity status for patients who were not treated with decompressive craniectomy – a GAM analysis. [file 13054_2023_4627_MOESM2_ESM.pdf]

Additional file 2. $PbtO_2$ in relation to cerebral physiological variables depending on the concurrent pressure or oxygen reactivity status for patients who were not treated with decompressive craniectomy – a GAM analysis

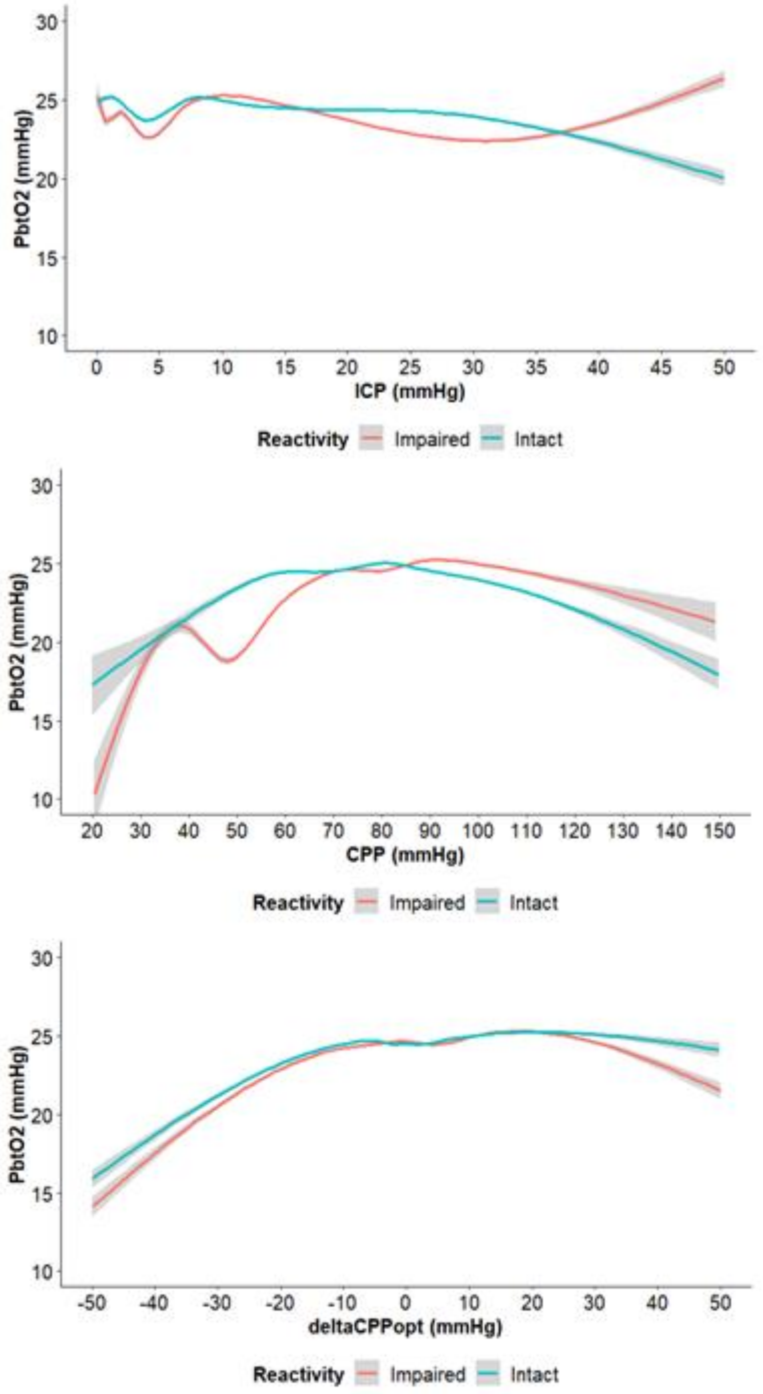

These GAMs were based on patients who did not undergo decompressive craniectomy (n=222), i.e. both those who received such treatment and those with unavailable data on decompressive craniectomy were excluded from these analyses. Impaired/intact reactivity was defined as PRx above/below 0.30.

CPP = Cerebral perfusion pressure. GAM = General additive model. ICP = Intracranial pressure. $PbtO_2$ = Partial brain tissue oxygenation. PRx = Pressure reactivity index.
